# Modification of 13X Molecular Sieve by Chitosan for Adsorptive Removal of Cadmium from Simulated Wastewater

**DOI:** 10.3390/ma10091101

**Published:** 2017-09-19

**Authors:** Yan Shi, Ken Sun, Lixin Huo, Xiuxiu Li, Xuebin Qi, Zhaohui Li

**Affiliations:** 1 School of Environmental and Municipal Engineering, North China University of Water Resources and Electric Power, Zhengzhou 450045, China; shiyan@ncwu.edu.cn (Y.S.); sunken@ncwu.edu.cn (K.S.); 18748975624@163.com (L.H.); 2 Henan Province key Laboratory of Water-Saving Agriculture, North China University of Water Resources and Electric Power, Zhengzhou 450045, China; lixiuxiu@163.com; 3 Farmland Irrigation Research Institute, CAAS, Xinxiang 453002, China; 4 Geosciences Department, University of Wisconsin—Parkside, Kenosha, WI 53144, USA

**Keywords:** cadmium, chitosan, modification, 13X molecular sieve, removal

## Abstract

Chitosan was used to modify a 13X molecular sieve to improve its cadmium removal capability. After being modified with 2% chitosan-acetate for 2 h at 30 °C, significant uptake of Cd^2+^ could be achieved. The uptake of Cd^2+^ on the modified 13X molecular sieve followed the Langmuir isotherms with a capacity of 1 mg/g. The kinetics of Cd^2+^ removal by modified 13X molecular sieve followed a pseudo second-order reaction, suggesting chemisorption or surface complexation. The Cd^2+^ removal with a sorbent dose of 2 g/L from an initial concentration of 100 μg/L reached more than 95% in 90 min. The equilibrium Cd^2+^ concentration was <5 μg/L, which meets the requirements of “Standards for Irrigation Water Quality” (GB5084-2005) (10 μg/L) and MCL and MCLG for groundwater and drinking water (5 μg/L) set by United States Environmental Protection Agency.

## 1. Introduction

Cadmium (Cd) is a major water pollutant. With a high toxicity, Cd can cause chronic intoxication with an incubation period from 10 to 30 years. The half-life of Cd in the human body also varies between 10 and 30 years. People can develop gastroenteropathy or even hepatopathy, lung cancer, kidney disease, with continuing intake of food or water contaminated with Cd [1,2,3,4]. Currently, the USEPA has set both the maximum contamination level (MCL) and maximum contamination level goal (MCLG) at 5 μg/L [5]. Although its concentration in unpolluted natural waters was usually below 1 μg/L [6], a maximum value of 100 μg/L was reported in Rio Rimao in Peru [7]. In addition, the presence of Cd as an impurity in zinc in galvanized pipes may have potential for drinking water contamination. A mean concentration of 1–26 μg/L was found in samples of potable water in Saudi Arabia [8].

Cd cannot be microbiologically degraded in the natural environment, and can only be dispersed, enriched, or converted between its various forms [9]. The migration process of Cd in water is mainly dependent on precipitation, complexation, and adsorption. Although natural zeolite has high cation exchange capacities (CEC), its internal channels of different sizes make the uptake of heavy metal ions more kinetically controlled [10,11]. In addition, natural zeolite may have other impurities that can make water purification less effective. Compared with natural zeolite, synthetic zeolite has simpler phases and compositions, with less impurity, which avoids secondary pollution. Its internal pore channels and holes would be of uniform size, which can be regulated by adjusting the synthesis conditions, as well as larger internal and external surface area, good physical and chemical stability, exchange, adsorption, and catalysis capacities make it a good candidate for water treatment [12,13]. In particular, the synthetic 13X zeolite molecular sieve (MS) has a cubic crystal system with 3D pore channels, which can provide a faster intracrystalline diffusion for adsorption and catalysis [14,15]. However, its uptake of cationic heavy metals may be low.

Containing a large number of amino and hydroxyl groups, chitosan has high hydrophilicity and can absorb heavy metals in wastewater [16,17,18]. Chitosan-modified natural zeolite can achieve good removal performance of heavy metals (mostly Cu(II), Fe(III), Mn(II), Zn(II)) from wastewater with high concentrations [19,20,21,22,23,24,25,26]. However, little research has been conducted into the treatment of wastewater with low Cd^2+^ concentrations using chitosan-modified 13X molecular sieves (CMS). Therefore, in this study, conditions were optimized for the modification of MS by chitosan, and the CMS was tested for the removal of low concentrations of Cd^2+^ from simulated wastewater, so that the Cd^2+^ concentration would meet the discharge standards of 10 μg/L set by the Standards for Irrigation Water Quality (GB5084-2005), and 5 μg/L of MCL and MCLG set by USEPA. The results also provide technical support for safe use of unconventional water in agriculture.

## 2. Materials and Methods

### 2.1. Materials

The 13X molecular sieve was off-white in color with a grain size of 2 to 3 mm, and was purchased from Zhengyuan Haoye Chemical Technology Co. (Tianjing, China). Chitosan (food grade with 95% deacetylation) was purchased from Zhengzhou Mingrui Food Ingredients, Co., Ltd. (Zhengzhou, China) Nitric acid, hydrochloric acid, sodium hydroxide, glacial acetic acid, and Cd^2+^ stock solution (1.000 g/L) were all of reagent grade or analytical pure and were purchased from Zhiyuan Chemical Technology Co. (Tianjing, China).

### 2.2. Preparation of CMS

The MS was washed with tap water several times to remove the impurities, and then washed with deionized water 2–3 times before being dried at 105 °C for modification. The MS was added to chitosan solutions of different concentrations (0.5–3.0%) under acidic conditions to increase the solubility of chitosan [16,18]. The mixture was shaken under a constant temperature of 30 °C at 120 rpm for 30, 60, 90, 120, 150, and 180 min, before being washed with deionized water to neutral condition and dried at 55 °C.

### 2.3. Cd^2+^ Adsorption Experiments

0.1 g of CMS was added into a conical flask, and then 100 mL of simulated wastewater containing 25, 50, 75, 100, 150, 200, and 250 μg/L of Cd^2+^ were added. The mixtures were stirred under constant temperature of 25 °C at 130 rpm for varying amounts of time. The supernatant was removed and the Cd^2+^ concentration was determined by a flame atomic adsorption spectrometry (FAAS) (WFX-210, Beifen-Ruili Analytical Instrument Co., Beijing, China). The amount of Cd^2+^ removed was calculated by the difference between the initial and equilibrium Cd^2+^ concentrations divided by the solid mass and multiplied by the liquid volume. The Cd^2+^ removal was calculated by the difference between the initial and equilibrium Cd^2+^ concentrations multiplied by 100. All experiments were done in triplicate, and the average and standard deviations are plotted in the figures.

### 2.4. Material Characterization

The material characterization was conducted using JSM-7500F scanning electron microscope (JEOL, Tokyo, Japan) equipped with energy dispersion spectrum (EDS) system. The BET analysis was performed at 120 °C after 60 min degassing with a N_2_ temperature of 77 K. The XRD was performed on a D8 Focus diffractometer from Bruker (Karlsruhe, Germany). Samples were run from 5 to 80° (2*θ*) with a scanning speed of 2°/min. The thermogravimetric analysis (TGA) was conducted using STA PT1600 Simultaneous Thermal Analysis manufactured by Linseis (Munich, Germany). The initial mass used was 10 mg, and the heating rate was 10 °C/min. The FTIR spectra were recorded on an IRAffinity FTIR spectrophotometer made by Shimadzu (Tokyo, Japan). Standard KBr pressing method was used for sample preparation and the scan was recorded from 500 to 4000 cm^−1^.

## 3. Results and Discussion

### 3.1. Effects of Different Modification Conditions on Cd^2+^ Removal

**Figure 1:**
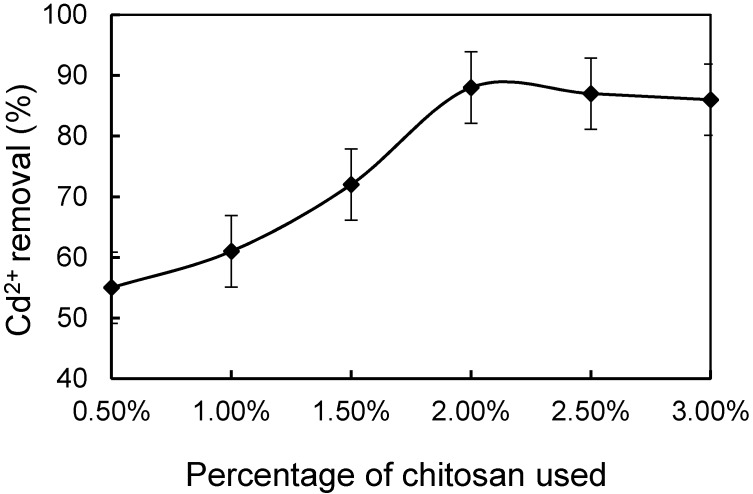
Effect of initial chitosan usage on Cd^2+^ removal.

The initial chitosan concentrations used for the preparation of CMS was assess first. When the chitosan concentration increased from 0.5% to 2%, the percentage of Cd^2+^ removal from simulated wastewater showed an increasing tendency. Beyond 2%, further increase of chitosan content did not affect Cd^2+^ removal much. At 2% chitosan modification, the Cd^2+^ removal by CMS reached up to 90% (Figure 1).

Chitosan belongs to the natural macromolecule amylose, which has similar properties to chitin and cellulose. When the chitosan concentration was over 2%, a gel-like condition was formed, which limited the contact between chitosan molecules and MS. Therefore, the optimal concentration of chitosan was set at 2% for the fabrication of CMS.

**Figure 2:**
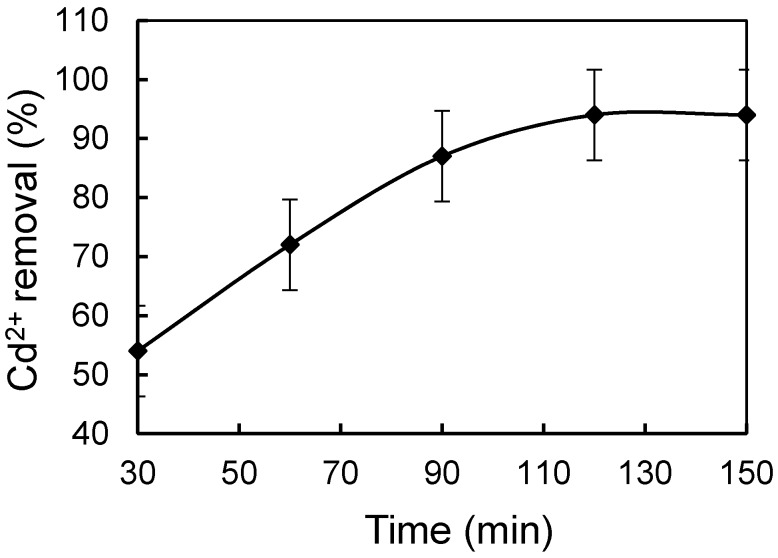
Effect of time of chitosan modification on Cd^2+^ removal.

The effect of time of chitosan modification on Cd^2+^ removal was assessed next, with Cd^2+^ removal peaking at 120 min (Figure 2). Thus, for the detailed Cd^2+^ removal experiment, the MS was modified by 2% chitosan for 120 min.

### 3.2. Material Characterization of MS and CMS

**Figure 3:**
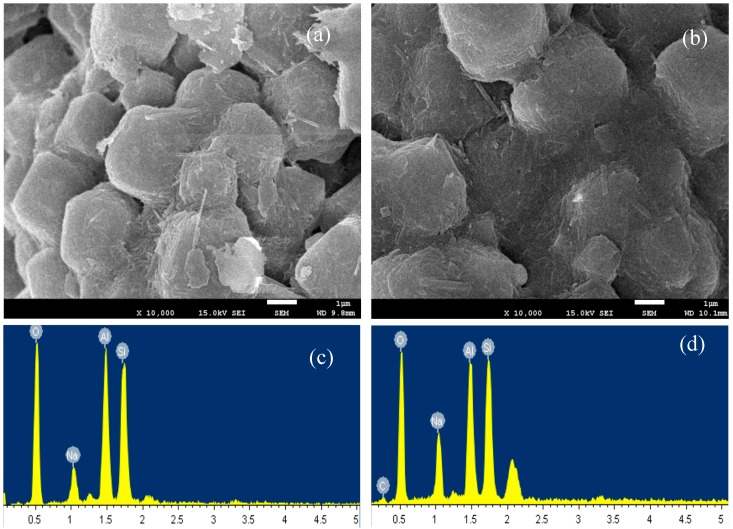
SEM images of MS (**a**) and CMS (**b**) and EDS images of MS (**c**) and CMS (**d**).

The MS showed granular particles with smooth surfaces and a particle size of 2–3 μm (Figure 3a). A similar texture was found for the CMS. The SEM images confirmed that the modification did not change the morphology or the internal structure much (Figure 3b). The EDS analyses showed that the MS was mainly composed of O, Na, Al, and Si elements (Figure 3c). The CMS showed an increase in C peak height, as well as the peak at 2.1 keV, attributed to the N element, confirming the uptake of chitosan on the MS (Figure 3d). Although the substitution of Al for Si in tetrahedron resulted in negative charges, the amount of substitution in the MS is relatively small. The BET surface area was 636 and 637 m^2^/g, the average pore size was 2.5 and 2.4 nm, and the pore volume was 0.35 and 0.35 cm^3^/g for MS and CMS, respectively. The MS results agreed well with a previous study [14]. The uptake of chitosan on MS resulted in a great increase in negative charges. Therefore, its uptake of Cd^2+^ was enhanced.

**Figure 4:**
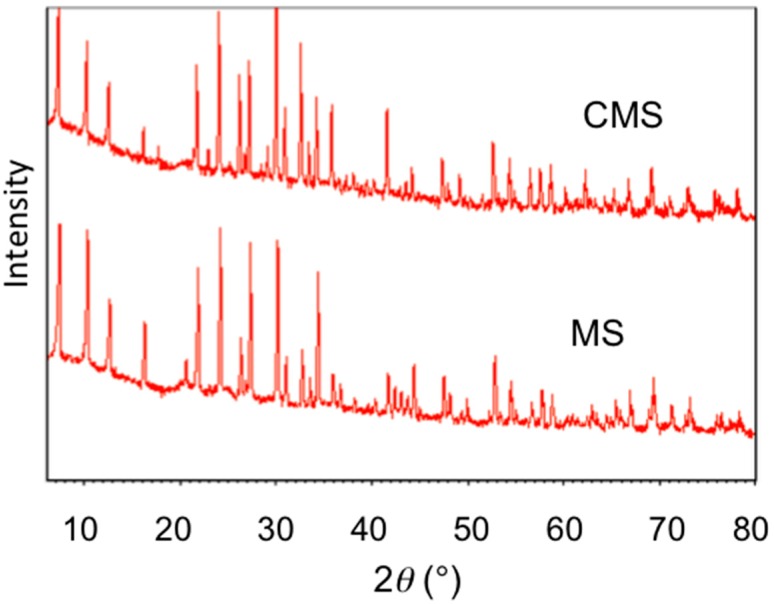
XRD patterns of MS and CMS.

**Figure 5:**
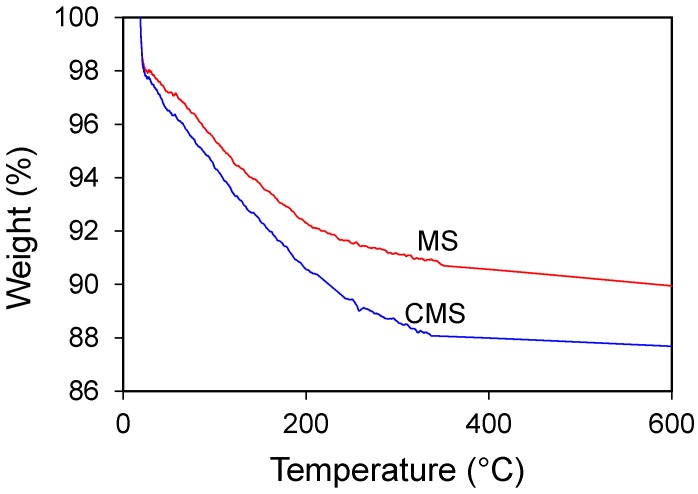
Thermogravimetric analysis of MS and CMS.

**Figure 6:**
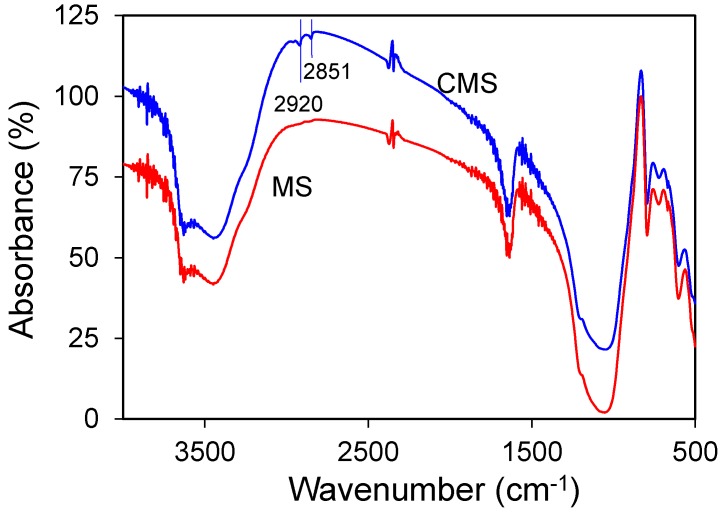
FTIR analysis of MS and CMS.

The X-ray diffraction (XRD) analyses before and after chitosan modification showed no change in material phases or structure (Figure 4), suggesting that the uptake of chitosan was on the external surfaces. The TGA showed slight different in mass loss (Figure 5). After chitosan modification, there was 2% more mass loss, which could be attributed to mass loss by chitosan. The FTIR analyses showed vibrations at 2850 and 2920 cm^−1^, attributed to the vibration of chitosan after modification (Figure 6). All these results showed successful modification of MS by chitosan. 

### 3.3. Effect of Different Physicochemical Conditions on Cd^2+^ Removal by CMS

**Figure 7:**
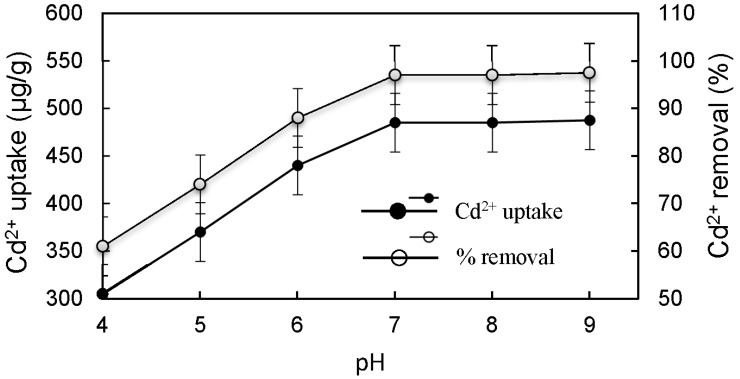
Effect of equilibrium solution pH on Cd^2+^ uptake and removal.

With an initial Cd^2+^ concentration of 100 μg/L, the Cd^2+^ uptake and removal varied with equilibrium solution pH (Figure 7). When the solution pH increased from 4 to 7, the Cd^2+^ removal increases quickly from 33.7% to 97.4%. This is mainly because of the fact that, in acid solutions, a high concentration of H ions is in competitive adsorption against Cd^2+^ ions, which impacts the adsorbing effect of the adsorbent. However, with further increase of solution pH beyond 7, the change in Cd^2+^ uptake did not change much (Figure 7). Therefore, the optimal pH value for maximal Cd^2+^ removal was determined to be 7.

**Figure 8:**
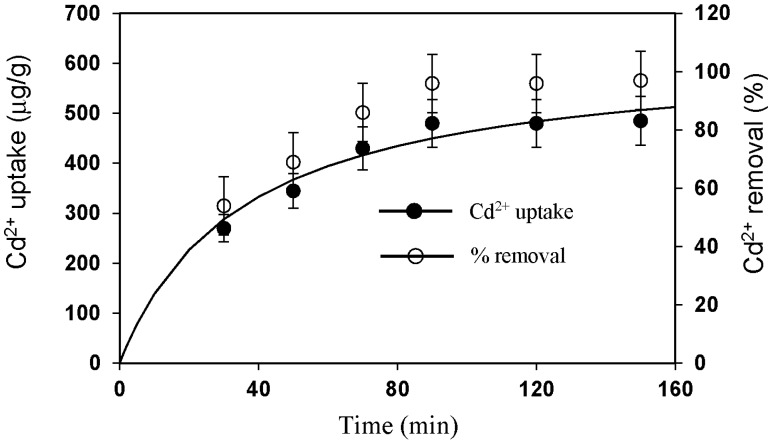
Kinetics of Cd^2+^ uptake and removal.

The kinetics of Cd^2+^ removal by CMS of 2% modification is shown in Figure 8. Equilibrium was able to be reached in 90 min. The data were fitted to several kinetic models, and the pseudo second-order model fitted the data best (r^2^ = 0.98). This has the formula:

(1) $$q_{t}=\frac{kq_{e}^{2}t}{1+kq_{e}t}$$

where *k* (g/μg-min) is the rate constant of adsorption, *q_e_* (μg/g) the amount of Cd^2+^ adsorbed at equilibrium, and *q_t_* (μg/g) is the amount of Cd^2+^ adsorbed on the surface of the adsorbent at any time, *t*. The fitted values are *q_e_* = 625 μg/g, *k* = 4.6 × 10^−5^ g/μg-min, and the rate constant is 17.8 μg/g-min.

**Figure 9:**
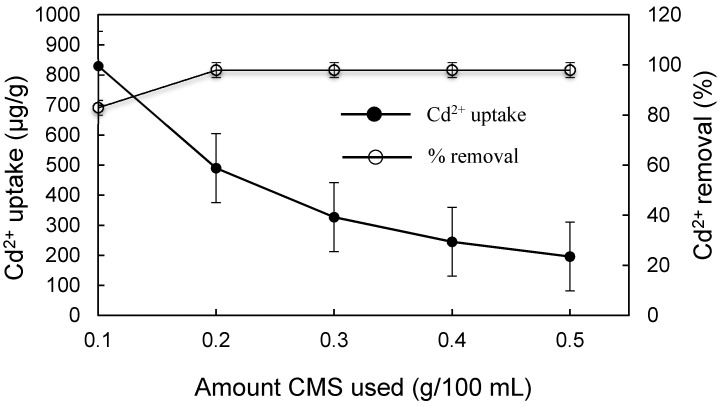
Effect of dosage of CMS on Cd^2+^ uptake and removal.

For the best application, the dosage of CMS was also evaluated. When the dosage of CMS was increased from 0.1 to 0.2 g, the cadmium removal quickly increased from 82.1% to 98.1% (Figure 9). The equilibrium Cd^2+^ was less than 2 μg/L, which meets the standard Cd^2+^ emission of 10 μg/L prescribed in the Standards for Irrigation Water Quality (GB5084-2005) and the MCL and MCLG of 5 μg/L for ground water and drinking water set by USEPA.

**Figure 10:**
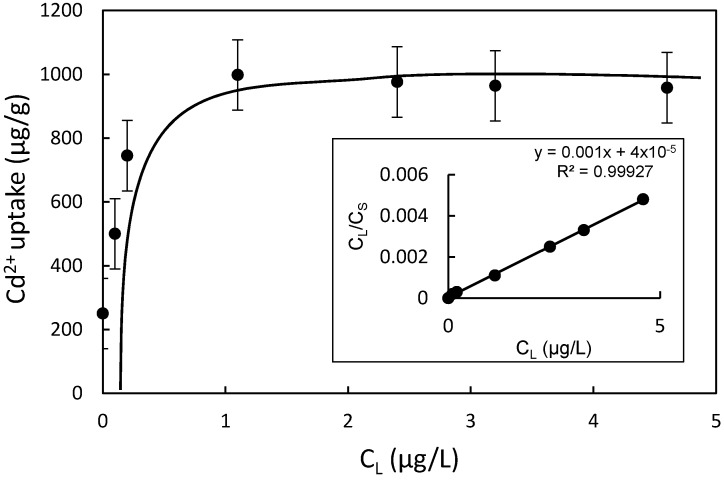
Uptake of Cd^2+^ on CMS. The inset is the linear Langmuir fit to the observed data.

The isotherm of Cd^2+^ uptake was fitted to both the Langmuir and Freundlich models, and the Langmuir model resulted in a much better fit for the experimental data, with a Cd^2+^ uptake capacity of 1 mg/g (Figure 10 and Table 1). The Langmuir sorption isotherm has the form:

(2) $$C_{S}=\frac{K_{L}S_{m}C_{L}}{1+K_{L}C_{L}}$$

where *C_S_* is the amount of Cd^2+^ adsorbed at equilibrium (μg/g), *S_m_* the apparent sorption capacity (μg/g), *C_L_* the equilibrium Cd^2+^ concentration (μg/L), and *K_L_* the Langmuir coefficient (L/μg). The Freundlich isotherm has the formula:

(3) $$C_{S}=K_{F}C_{L}^{n}$$

where *K_F_*, *n*, and Freundlich constants reflect the adsorption capacity and intensity, respectively. The agreement with the Langmuir model suggested the monolayer adsorption of Cd^2+^ on CMS surfaces, confirming the effectiveness of chitosan modification.

The Cd^2+^ uptake and removal by CMS was compared to that by MS without chitosan modification. A reduction of almost 80 times in equilibrium Cd^2+^ concentration, or a 10-fold increase in Cd^2+^ uptake was achieved by CMS in comparison to MS alone (Table 2).

Preliminary studies showed that the Cd^2+^ uptake and removal was endothermic with the ΔG, ΔH, and ΔS values of 7.4 kJ/mol, 50.6 kJ/mol, and 0.2 kJ/(mol·K), respectively.

The chitosan molecules have large amounts of amino and hydroxyl groups. These functional groups could form complexation with divalent metal cations. Previous results showed that one Cu(II) cation complexed with two amino and groups of chitosan [22]. The agreement of the pseudo-second order kinetic data also pointed to chemisorption [18,19].

## 4. Conclusions

1. The optimal condition for the preparation of CMS was mixing 2% chitosan solution with MS for 2 h.
2. SEM and XRD results showed no change in crystal morphology of MS after modification. However, the increase in C and N contents in EDS spectra, the increase in 2% of mass loss in TGA analysis, and the presence of 2850 and 2920 cm^−1^ bands in FTIR analyses confirmed chitosan uptake on MS after modification.
3. The static single factor experiment results showed that under the condition of room temperature, pH of 7, vibration adsorption time of 90 min, and adsorbent dosage of 2 g/L, the Cd^2+^ removal from simulated wastewater with an initial concentration of 100 μg/L was over 96% when using CMS as the adsorbent, which meets the standard Cd^2+^ discharge prescribed in the Standards for Irrigation Water Quality (GB5084-2005) and the MCL and MCLG for groundwater and drinking water standards set by USEPA.
4. The adsorption process of lower concentration of Cd^2+^ in water by CMS fitted with the Langmuir adsorption isotherm model well, with a saturated adsorption capacity of 1 mg/g.

## Figures and Tables

**Table 1:** Langmuir and Freundlich parameters for Cd^2+^ uptake on CMS.

Langmuir Constant			Freundlich Constant		
*S_m_* (μg/g)	*K_L_* (L/μg)	R^2^	logk	1/*n*	R^2^
1000	25	0.9993	2.70	0.534	0.47

**Table 2:** Comparison of Cd^2+^ removal by MS and CMS.

Sample	C_L_ (μg/L)	Removal (%)	C_S_ (μg/g)
MS	82.1	17.9	89.5
CMS	1.1	98.7	985
